# A Modified Two-Layer Suture Technique for Transperitoneal Laparoscopic Partial Nephrectomy: Single-Center Clinical Experience

**DOI:** 10.3389/fsurg.2021.761090

**Published:** 2022-02-01

**Authors:** Yang Jin, Hui Xiong, Qinghua Xia, Qi Zhang

**Affiliations:** ^1^Medical Research Center, Hospital Affiliated to Binhai University, Qingdao, China; ^2^Department of Urology, Shandong Provincial Hospital Affiliated to Shandong First Medical University, Jinan, China

**Keywords:** glomerular filtration rate, suture, laparoscopic partial nephrectomy, warm ischemic time, blood urea nitrogen

## Abstract

**Purposes:**

This study aims to evaluate the feasibility and efficacy of a modified two-layer suture method during laparoscopic partial nephrectomy (LPN) by a comparative analysis with the traditional two-layer suture.

**Methods:**

A total of 60 LPN patients were enrolled in this study, of which 30 patients received the modified two-layer suture method and the remaining 30 patients underwent the traditional two-layer suture. Then, surgical characteristics including operative time, warm ischemic time (WIT), estimated blood loss (EBL), and glomerular filtration rate (GFR) were recorded. Finally, univariable and multivariable linear regression analyses were used to evaluate the correlations of tumor characteristics, suture methods, and postoperative renal function.

**Results:**

There was no significant difference between the two suture groups with respect to patient and tumor characteristics, postoperative creatinine level, and blood urea nitrogen (BUN) level. The modified suture group showed a significantly shorter clamping time and a less GFR level reduction than the traditional two-layer suture group (15 vs. 23 min; 42.32 ± 9.48 vs. 27.07 ± 7.88; *p* < 0.05). Additionally, the modified two-layer suture was an independent factor that influenced the clamping time and the level of GFR reduction.

**Conclusion:**

The modified two-layer suture method is feasible and effective for LPN.

## Highlights

- A modified two-layer suture technique was presented.
- A modified two-layer suture reduced WIT during LPN.
- A modified two-layer suture was feasible for LPN.

## Introduction

Renal cell carcinoma (RCC) is a common malignant tumor in the genitourinary system with a higher incidence around the world (1). Surgical excision is an optimal choice for treating RCC over the last few decades. Partial nephrectomy (PN), a nephron-sparing surgery, has been proven to be a successful surgical strategy for T1 tumors (2). Although no differences between PN and radical nephrectomy (RN) in overall survival rates are observed, PN exhibits more favorable functional outcomes than RN, such as better renal function preservation and less surgical complications (3, 4). Notably, laparoscopic PN (LPN), as a minimally invasive technique, has currently obtained extensive attention by increasing experienced surgeons. Moreover, existing evidence has demonstrated that the practicability and reliability of LPN are based on multiple outcome measures, including margin negativity, reduced warm ischemic time (WIT), preserving the maximum amount of renal parenchyma, and decreased risks of postoperative bleeding, and urinary leakage (5, 6). Among them, WIT plays a crucial role in predicting short-term renal function after surgical procedures (7). Therefore, multiple modified operation methods such as zero ischemia or off-clamp technique for LPN have been presented to reduce WIT duration, thereby minimizing the loss of renal function after LPN. However, these surgical procedures appear to be effective for small or peripheral RCC (8, 9). For larger tumors, the clinically contemporary techniques involve hilar clamping, which may cause an ischemic and long-term decline in kidney function (10).

Notably, the efficiency and quality of wound suturing are not only strongly correlated with WIT, but it is also an effective measure to control postoperative hemorrhage and urine leak. Currently, a series of innovative suturing methodologies, such as single-layer and two-layer sutures, were introduced to suture wound in the kidney. Previous results show that the single-layer access technique reduces the ischemic duration and the total operation time but increases the risk of postoperative complications (e.g., bleeding and urinary leak) (11). Two-layer suturing is a reliable suture procedure during LPN, which contains the inner layer to suture the base and the outer layer to suture the edge of renal parenchyma (12). Besides, such a suture method involves a clamp for the renal artery, creating reduced blood flow, and decreased risks of intraoperative hemorrhage (13, 14). However, compared with the single-layer suture, there was a longer clamping time, WIT duration, and operative time in the two-layer suture method. Therefore, the improvement of the technical operation still needs to be redesigned and improved.

In the present study, we present a modified two-layer suturing method to reduce WIT. Besides, we performed a comparative analysis to assess the feasibility and safety of this novel suture technique during transperitoneal LPN.

## Methods

### Patient Selection

A total of 60 consecutive patients with renal tumor in Shandong Provincial Hospital Affiliated to Shandong First Medical University between January 2018 and December 2019 were recruited in this study. These 60 patients were randomly divided into two groups, 30 patients underwent a modified two-layer suturing during LPN. Meanwhile, the remaining 30 patients were treated by the conventional two-layer suture method. The data from all patients were collected prospectively. All surgeries were performed by the same surgeon (QZ). The inclusion criteria for all subjects were as follows: (1) all patients were diagnosed with unilateral and solitary renal tumors according to the enhanced CT scanning preoperatively, and their contralateral kidneys were normal, all tumors were proven to be RCC by pathology after the operation. (2) The glomerular filtration rate (GFR) of the contralateral kidney before the surgical operation was within the normal range value. Those patients who suffered from upper urinary tract obstruction caused by other pathogenic factors were excluded. This study was approved by the Shandong Provincial Hospital Ethics Committee, all methods in this research were carried out in accordance with the relevant guidelines of RCC treatment, and all patients signed written informed consent.

All patients were subjected to routine blood examinations and renal function tests [creatinine, blood urea nitrogen (BUN)] preoperatively and postoperatively. One day before the surgery and one day after the surgery, all patients received renal scintigraphy examination to measure the GFR level. The demographic characteristics (sex, age, and body mass index) of patients were collected. Besides, tumor characteristics, including tumor size, location (anterior, posterior, and striding), T stage (T1a, T1b), growth pattern (exophytic, mesophytic, and endophytic) were also recorded and analyzed. Herein, the striding tumor was defined as the lesion located on both the anterior and posterior sides of the kidney as previously reported (15). Besides, the exophytic and endophytic tumors were respectively considered to be the lesions extending >60 and <40% from the kidney surface, whereas the mesophytic tumor was the lesion that extends between 40 and 60% from the natural surface of the kidney (15).

### Surgical Technique

The transperitoneal approach was used for all surgical procedures. The first trocar port was placed at 2 cm below the crossing between the midclavicular line and the costal margin. The second port was made at the lateral border of rectus abdominis, and then the laparoscope was inserted through piercing with a 10-mm trocar. Subsequently, the third port is placed at the junction between the costal margin and anterior axillary line. Notably, the location of the trocar ports can be adjusted moderately according to the tumor site. In some cases, an auxiliary port was created at the intersection of 3–5 cm below the umbilicus and midclavicular line.

**Figure 1:**
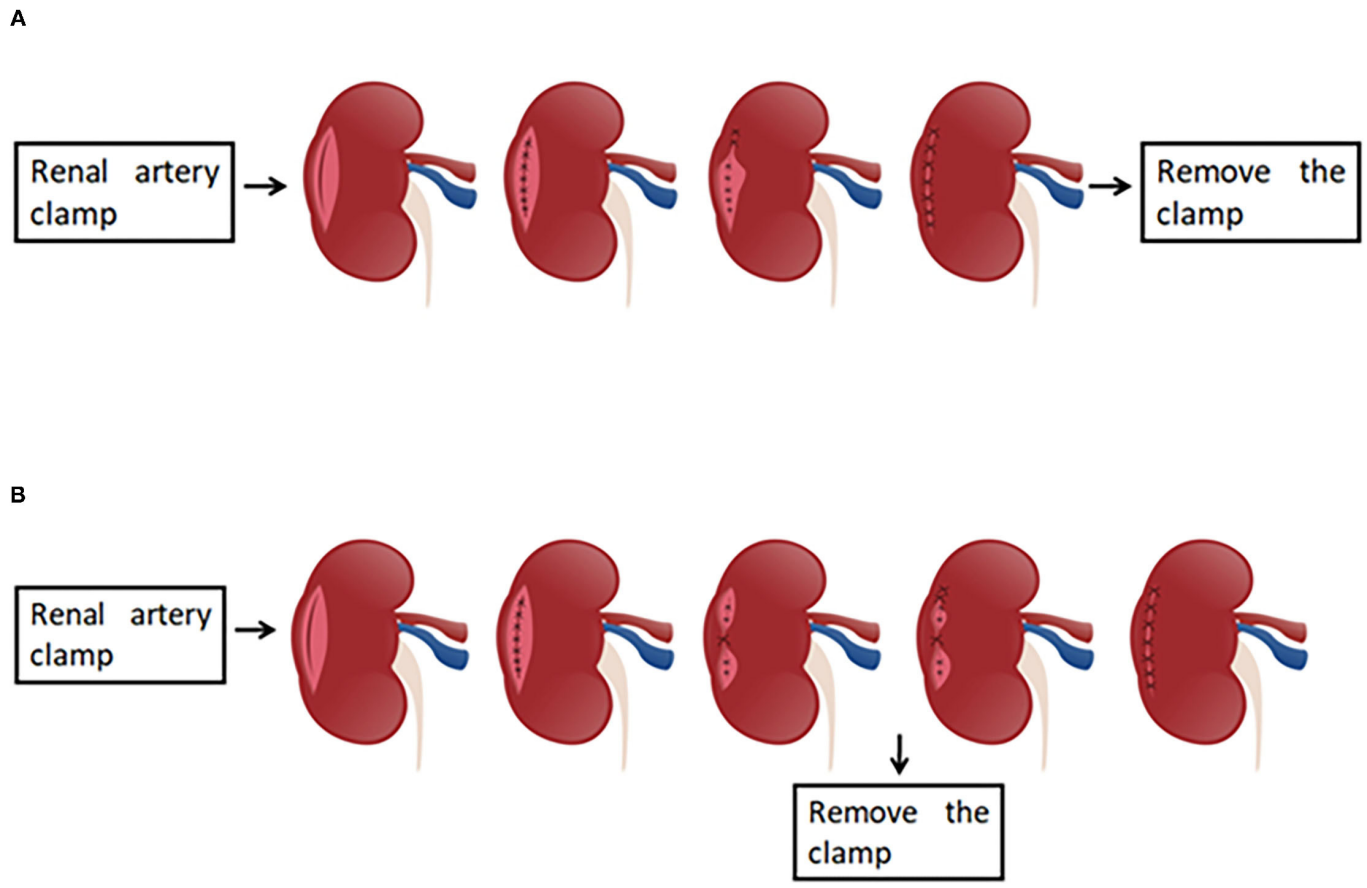
The presentation of the traditional two-layer suture and modified two-layer suture. **(A)** The graphical representation of the traditional two-layer suture. After the renal artery is clamped, the suture is done in the base (inner layer), and subsequently the sutures are run from one end of the outer layer to the other end, the clamp is removed at last. **(B)** The presentation of the modified two-layer suture. The steps are same as those in traditional suture method until the inner layer suture is finished. When the suture for outer layer is performed, the first suture is initiated at the central point of the outer layer, and the tail of the suture was secured by Hem-o-Lok clips. Consequently, one side of the outer layer was attached closely to another side so that the potential hemorrhagic spots were pressed. Then, the vascular clamp is removed to restore kidney perfusion, and the sutures are continuously run from top to bottom.

**Figure 2:**
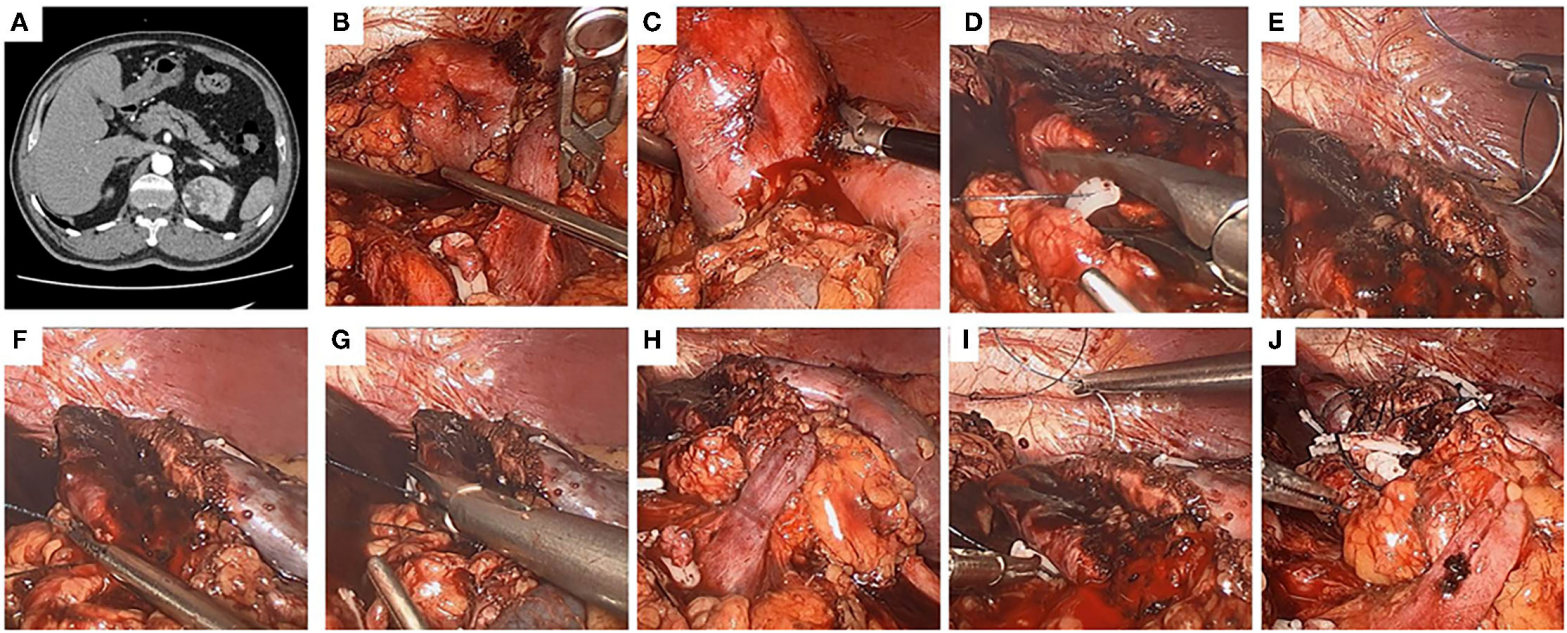
The use of the modified two-layer suture in a left kidney. A left-kidney, 4.1-cm-diameter, mesophytic tumor striding both anterior and posterior sides, and the modified two-layer suture technique was performed. **(A)** Crosssection of computed tomography (CT) scan. **(B)** The renal artery was clamped. **(C)** The tumor was removed by a scissors. **(D)** The inner layer was closed by continuous suture, and the tail of the suture was secured by a Hem-o-Lok. **(E,F)** The modified two-layer suture was used to close the outer layer, the first suture began at the central point of the outer layer. **(G)** Both the inserting and removing points of the first suture were secured by Hem-o-Lok clips, so that the two sides of the outer layer were attached closely to each other. **(H)** The clamp was removed to restore kidney perfusion. **(I)** The sutures were continuously run from one end to the other end in the outer layer. **(J)** The kidney wound was closed.

**Figure 3:**
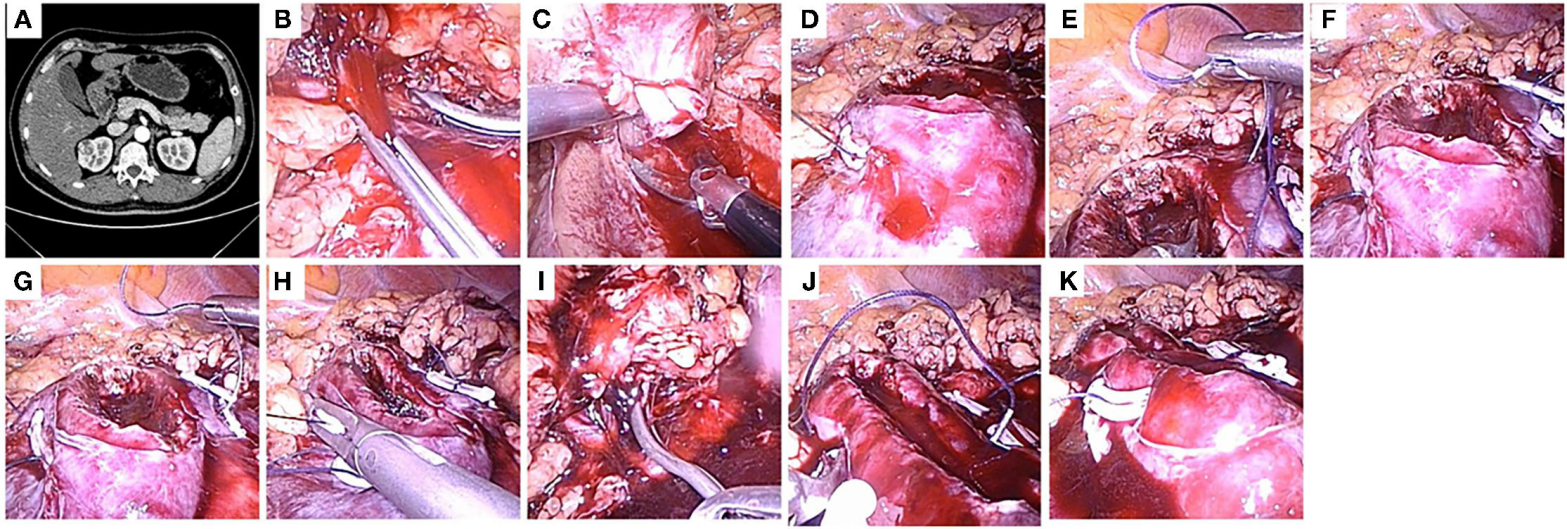
The use of the modified two-layer suture in a right kidney. **(A)** A 2.8-cm-diameter, mesophytic tumor located at anterior side of a right kidney, and the modified two-layer suture technique was performed. A crosssection of computed tomography (CT) scan. **(B)** The renal artery was clamped. **(C)** The tumor was removed by a scissors. **(D)** The inner layer was closed by continuous suture, and the tail of the suture was secured by a Hem-o-Lok. **(E–G)** The modified two-layer suture was used to close the outer layer, the initial figure-of-eight suture was begin at the central point of the outer layer. **(H)** The inserting and removing points of the sutures were secured by Hem-o-Lok clips, so that the two sides of the outer layer were attached closely to each other. **(I)** The clamp was removed to restore kidney perfusion. **(J)** The sutures were continuously run from one end to the other end in the outer layer. **(K)** The kidney wound was closed.

After the working space inside the abdominal cavity was established, the Gerota fascia was incised, which provided easy access to the renal artery. Next, the renal artery clamping technique was adopted and the tumor was removed along the surface which was about 2 mm from the tumor capsule. For wound suturing, 2-0 and 3-0 V-loc bared sutures anchored with Hem-o-Lok clips at the tail end were used for the repair of renal parenchyma defect. The running suturing with 3-0 V-loc was done in the inner layer of renal parenchyma. The sutures were run from one end of the inner layer to the other and finally secured by Hem-o-Lok clips at the end position. For traditional suture, the continuous suture was also performed for the edge repair of the renal remnant (the outer layer of renal parenchyma). Moreover, the renal artery clamp would be removed until two layers wound closure, which was entirely different from our new outer layer suturing. For the modified suturing method, the initial first suture or a figure-of-eight suture was initiated at the central point of the outer layer, and the tail of the suture was secured by Hem-o-Lok clips. Consequently, one side of the outer layer was attached closely to another side so that the potential hemorrhagic spots were pressed. Afterward, the vascular clamp was removed to restore kidney perfusion. Finally, the sutures were continuously run from top to bottom throughout the border of the outer layer (Figures 1–3).

### Postoperative Assessment and Follow-Up

Surgical characteristics (operative time, clamping time, estimated blood loss (EBL), and the length of hospital stay) were recorded. Postoperative complications were classified based on Clavien's method. The GFR examination of all patients was performed 1 day after the operation, and functional outcomes (recurrence rate and mortality) were evaluated by the CT scan after the follow-up of 1, 3, and 6 months.

### Statistical Analysis

In this study, Student's *t*-test and the chi-squared test were respectively used for the comparative analysis of continuous variables and categorical variables. The relationships between tumor characteristics (size, location, and growth pattern) and the option of the suturing method were assessed by univariable and multivariable logistic regression analyses. Besides, we used univariable and multivariable linear regression analyses to evaluate the correlations of tumor characteristics, suture methods, and postoperative renal function. The value of *p* < 0.05 shows a statistically significant difference in the comparison.

## Results

### Baseline Demographic Data and Preoperative Information

Patients' baseline demographic and preoperative tumor characteristics were summarized in Table 1. Overall, there were 30 patients (20 men and 10 women) in the modified suture group and 30 patients (18 men and 12 women) in the traditional suture group. There were no significant differences between the two groups with respect to all comparable parameters. The mean age of the patients was 58.4 years with a range from 34 to 74 years. There were 34 (56.7%) patients in the T1a stage and 26 (43.3%) patients in the T1b stage. The malignant lesions were primarily located on anterior or posterior sides of the kidney and considered as exophytic (43.3%) or endophytic tumors (16.7%). Herein, the striding tumor, which is defined as the lesion located on both the anterior and posterior sides of the kidney, accounted for 23.3%.

**Table 1:** Overall patients baseline demographic data and preoperative information.

**Variables**	**Traditional suture group**	**Novel suture group**	**χ2/t/Z**	***P***
Patients, no.	30	30		
Male	18 (60.00%)	20 (66.67%)	0.144	0.705
Female	12 (40.00%)	10 (33.33%)		
Age, year, median (range)	58.8 (34–72)	58 (36–74)	0.021	0.983
Tumor size, cm, median (range)	4.32 (2.3–6.5)	4.39 (2.2–6.7)	4.36 (2.2–6.7)	
≤4(T1a)	16 (53.33%)	18 (60.00%)	0.136	0.713
4–7(T1b)	14 (46.67%)	12(40.00%)		
Tumor location, no. (%)				
Anterior	14 (46.67%)	12 (40.00%)	0.220	0.896
Posterior	10 (33.33%)	10 (33.33%)		
Striding	6 (20.00%)	8 (26.67%)		
Growth pattern, no. (%)				
Exophytic	12 (40.00%)	14 (46.67%)	0.277	0.871
Mesophytic	12 (40.00%)	12 (40.00%)		
Endophytic	6 (20.00%)	4 (13.33%)		
Preoperative GFR level, ml/min (affected side), mean ± SD	48.64 ± 3.78	47.78 ± 2.91	0.677	0.504
Preoperative creatine level, ml/min (affected side), mean ± SD	67.26 ± 9.7	68.55 ± 8.78	0.364	0.719
Preoperative BUN level, ml/min (affected side), mean ± SD	4.45 ± 0.91	4.25 ± 1.22	0.560	0.580

*GFR, glomerular filtration rate; SD, standard deviation; BUN, blood urea nitrogen*.

### Surgical Outcomes

In this study, 30 patients were all successfully approached by the modified suture method without the need to block the renal artery again. Among them, surgical wound bleeding occurred in one patient when the renal artery clamp was removed. Here, an aspirator-assisted laparoscopic approach was used during the running suturing. Accordingly, the surgical wound-surface showed no hemorrhage after the intracorporeal parenchymal suturing was completed.

Surgical outcomes are presented in Table 2. Although there was a shorter operative time and a lower EML in a modified two-layer suture group than that in the conventional suture group, no significant statistical difference was observed (operative time: *p* = 0.653; EBL: *p* = 0.771). Besides, there were no significant differences between the two groups with respect to postoperative creatine and BUN level (*p* > 0.05). However, there was a significant shorter clamping time (15 min with a range from 9 to 22 min vs. 23 min with a range from 14 to 34 min; *p* = 0.002), a higher level of postoperative GFR (34.81 ± 3.66 vs. 25.15 ± 3.75; *p* < 0.001), and a less GFR level reduction (27.07 ± 7.88 vs. 42.32 ± 9.48; *p* < 0.001) in the modified suture group than that in the traditional two-layer suture group. One patient in each group underwent hematuria (Clavien grade 1 complication) that was resolved after 2 weeks of bed rest without further intervention. Meanwhile, one patient had arterial and venous bleeding from the parenchymal defect and required blood transfusion (Clavien grade 2 complication) in the traditional suture group. Blood transfusion was indicated when the intraoperative hemoglobin level was <10 g/dl. The hemorrhage was controlled with suturing. No patient had positive surgical margins. No patient was converted to open surgery or RN. The median hospital stay was 7 days. All patients were followed up for a median period of 6 months postoperatively, with no postoperative hemorrhage requiring intervention, renal dysfunction, or arteriovenous fistula.

**Table 2:** Surgical characteristics.

**Variables**	**Traditional suture group**	**Novel suture group**	**t/Z**	***P***
Operating time, min, mean ± SD	103.6 ± 18.1	100.6 ± 16.8	0.455	0.653
Clamping time, min, median (range)	23 (14–34)	15 (9–22)	3.173	0.002
EBL, ml, median (range)	168 (50-650)	165 (80–500)	0.294	0.771
Postoperative GFR level, ml/min (affected side), mean ± SD	25.15 ± 3.75	34.81 ± 3.66	6.898	<0.001
Postoperative GFR reduction, % (affected side), mean ± SD	42.32 ± 9.48	27.07 ± 7.88	6.450	<0.001
Postoperative creatine level, μmoI/L, mean ± SD	69.41 ± 11.88	70.1 ± 10.57	0.163	0.871
Preoperative BUN level, nmoI/L, mean± SD	4.34 ± 0.77	4.31 ± 0.8	0.090	0.929
Complications, no.				
Grade 1 (hematuria)	1 (6.67%)	1 (6.67%)	-	1.000
Grade 2(major hemorrhage requiring transfusion)	1 (6.67%)	0	-	1.000
Grade 3a (postoperative hemorrhage requiring intervention)	0	0	-	-
Positive surgical margin, no.	0	0	-	-

*GFR, glomerular filtration rate; SD, standard deviation*.

### Univariable and Multivariable Analyses

Our univariable and multivariable analyses suggested that tumor size, growth pattern, and location were all not independent factors affecting the feasibility of two different parenchyma suturing techniques (*p* > 0.05; Table 3). Subsequently, anatomical features, intraoperative variables, and preoperative GFR levels have integrated the analyses of GFR reduction and clamping time. As indicated in Table 4, tumor size, growth pattern, the location was not associated with GFR reduction (*p* > 0.05; Table 4). However, the novel suture method was an independent factor that influenced the level of GFR reduction (OR=-17.594; 95%CI:−25.747 to−9.442; *p* < 0.001; Table 4). Although clamping time was related to GFR reduction, it was not an independent predictor (univariable analysis: OR = 15.647; 95% CI: −6.632 to 24.662; *p* = 0.001; multivariable analysis: OR = 6.409; 95% CI: −2.444 to 14.542; *p* = 0.153; Table 4). Similarly, tumor characteristics were not independent factors affecting GFR reduction (*p* > 0.05; Table 5). Notably, the modified suture was significantly correlated with clamping time according to the multivariable analysis (OR = −7.117; 95% CI: −11.332 to −2.903; *p* = 0.002; Table 5).

**Table 3:** The influence of tumor characteristics on the choice of suture method.

**Variables**	**Group 1**	**Group 2**	**Univariable analysis OR (95%CI)**	***P*** **value**	**Multivariable analysis OR (95%CI)**	***P*** **value**
Size, cm						
≤4	16	18	-	-	-	-
4–7	14	12	0.763 (0.179–3.241)	0.784	0.337 (0.044–2.606)	0.297
Growth pattern						
Exophytic	12	14	-	-	-	-
Mesophytic	12	12	1.750 (0.215–14.224)	0.601	3.014 (0.294–30.931)	0.353
Endophytic	6	4	1.500 (0.181–12.459)	0.707	1.923 (0.218–16.940)	0.556
Tumor location						
Anterior	14	12	-	-	-	-
Posterior	10	10	0.643 (0.101–4.097)	0.640	0.205 (0.014–2.995)	0.247
Striding	6	8	0.750 (0.107–5.238)	0.772	0.325 (0.027 3.946)	0.378

*Group 1: Traditional suture group; Group 2: Novel suture group*.

*OR, odds ratio; CI, confidential interval*.

**Table 4:** Univariable and multivariable associations with reduction in glomerular filtration rate.

**Variables**	**Univariable analysis OR (95%CI)**	***t***	***P*** **value**	**Multivariable analysis OR (95%CI)**	***t***	***P*** **value**
Size, cm						
≤4	-		-	-	-	-
4–7	5.548 (−4.972–16.068)	1.080	0.289	3.984 (−5.229–13.196)	0.899	0.379
Growth pattern						
Exophytic	-	-	-	-	-	-
Mesophytic	2.183 (−8.645–13.010)	0.413	0.683	−1.933 (−10.201–6.336)	−0.486	0.632
Endophytic	0.296 (−13.980–14.572)	0.043	0.966	−3.061 (−13.652–7.530)	−0.601	0.554
Tumor location						
Anterior	-	-	-	-	-	-
Posterior	4.652 (−6.490–15.793)	0.855	0.400	6.801 (−1.515–15.117)	1.701	0.104
Striding	−0.531 (−13.109–12.047)	−0.086	0.932	1.204 (−10.867–13.276)	0.207	0.838
Suture method						
Traditional suture	-	-	-	-	-	-
Novel suture	−21.251 (−28.001–14.502)	−6.450	<0.001	−17.594 (−25.747–9.442)	−4.488	<0.001
Clamping time						
≤20	-	-	-	-	-	-
>20	15.647 (6.632–24.661)	3.555	0.001	6.049 (−2.444–14.542)	1.481	0.153
Preoperative GFR level, ml/min (affected side), mean ± SD	3.962 (−1.623–9.547)	1.453	0.157	2.327 (−1.540–6.194)	1.251	0.225

*GFR, glomerular filtration rate; OR, odds ratio; CI, confidential interval; SD, standard deviation*.

**Table 5:** Univariable and multivariable associations with clamping time.

**Variables**	**Univariable analysis OR (95%CI)**	***t***	***P*** **value**	**Multivariable analysis OR (95%CI)**	***t***	***P*** **value**
Size, cm						
≤4	-	-	-	-	-	-
4–7	0.643 (−3.960–5.245)	0.286	0.777	0.070 (−5.534–5.674)	0.026	0.980
Growth pattern						
Exophytic	-	-	-	-	-	-
Mesophytic	0.425 (−4.235–5.084)	0.187	0.853	0.525 (−4.405–5.455)	0.221	0.827
Endophytic	−0.692 (−0.6815–5.432)	−0.231	0.819	−1.018 (−7.471–5.435)	−0.327	0.747
Tumor location						
Anterior	-	-	-	-	-	-
Posterior	−1.979 (−6.764–2.806)	−0.847	0.404	−2.171 (−7.138–2.795)	−0.907	0.374
Striding	−0.382 (−5.781–5.017)	−0.145	0.886	−0.565 (−7.920–6.789)	−0.159	0.875
Suture method						
Traditional suture	-	-	-	-	-	-
Novel suture	−7.071 (−10.729–3.414)	3.960	<0.001	−7.117 (−11.332–2.903)	−3.502	0.002
Preoperative GFR level, ml/min (affected side), mean ± SD	0.299 (−2.185–2.783)	0.246	0.807	−0.107 (−2.465–2.252)	−0.094	0.926

*GFR, glomerular filtration rate; OR, odds ratio; CI, confidential interval; SD, standard deviation*.

## Discussion

Laparoscopic partial nephrectomy represents a favored surgical strategy for high-volume experienced urological surgeons (16, 17). Kidney wound suture is a challenging task of this procedure and strongly correlated with postoperative outcomes (18). Accordingly, several technical innovations in suturing mode have upgraded the efficiency of the intracorporeal suture during an acceptable WIT window, especially for minimally invasive PN (12, 19, 20). Herein, a modified two-layer suture method was adopted in LPN based on a transperitoneal approach. This novel surgical technique dramatically reduced the clamping time, WIT, and improved postoperative kidney function compared to the traditional suture procedure.

Conventional renal suturing involves two layers (the base and the edge of parenchyma) suturing in a running fashion. Consequently, the renal artery is released until the completion of suturing. For the modified suture method, the inner layer suture runs continuously as a traditional two-layer suturing. The major difference is that the first suture is started at the middle line of the outer layer after the inner layer is sutured, and the two sides of the outer layer are attached to each other closely, which allows the removal of the clamp and renal reperfusion. Obviously, the clamping time is significantly shortened by this new suture technique, ensuring sufficient blood supply to the normal parenchyma. Additionally, statistical data suggested that there was a remarkably shorter clamping time for larger kidney tumors (the diameter > 4 cm), and the modified suture was significantly correlated with clamping time. Moreover, no patients underwent major hemorrhage requiring transfusion in the modified suture group, whereas there was one case of hemorrhage in the traditional suture group. Consistent with this, Zhang et al. previously pointed out that the traditional suturing method was unsuitable for large tumors due to numerous factors, including the general damage, longer WIT, and surgical complications such as hemorrhage after clam removal (21).

In recent years, different techniques have been proposed to reduce clamping time and WIT. Introini et al. revealed that the clampless sutureless LPN was safe and effective for renal tumors with low nephrometry score (tumor with size≤4 cm, intraparenchymal depth≤1.5 cm, renal nephrometry score between 4 and 6, and no close contact with the collecting system) (22). Ota et al. adopted a PN technique using soft coagulation without renorrhaphy (the mean tumor size was 28.2 mm) (23). Li et al. proposed a “pressure cooker” sutureless LPN method, which involves the covering of renal defect layer by layer with FloSeal, Tisseel, and a fat pad after monopolar coagulation (24). The above methods are suitable for the treatment of T1a renal tumor; however, the rigid instrumentation and tumor size selection limited the application and promotion. Compared with the traditional suture, the modified two-layer suture method in our study could significantly reduce the WIT in the operation for T1 renal tumor. Besides, the short learning curve and the need for general operating instruments can make the widespread of this suture much easier. The blood flow was interrupted when the renal artery was clamped, leading to WIT. WIT has been widely considered as a critical standard for postoperative renal function recovery. However, an ideal WIT threshold is a long-term controversy. Many researchers argued that functional outcomes were similar between off-clamp and on-clamp groups under the constraint of WIT (25, 26). In contrast, others believed that any effort should be taken to minimize the warm ischemia, and a shorter clamping time is a prerequisite for the successful recovery of renal blood flow and the postoperative kidney function (27, 28). Jeldres et al. evaluated several potential variables affecting renal construction following PN in patients with kidney tumors and found that the clamping time and blood loss were two independent predictors (29). Our work reported that a significant decrease in reduced GFR level at 1 day after surgery in our novel suture group was observed. Furthermore, the clamping time was markedly associated with GFR reduction. Accumulating evidence has demonstrated that early postoperative GFR can potentially predict the preserved kidney function outcomes (30, 31). For example, Erdem and colleagues highlight that the lowest estimated GFR was in the early postoperative period (about 1–3 days), which showed a strong predictive value for nephron-sparing advantage of self-retaining barbed suture for inner layer (32). However, the influence of GFR reduction on long-term kidney function still merits discussion. Taken together, we suggest that WIT was significantly reduced during the novel two-layer suturing, which encourages a lower decline of early postoperative renal function, and we especially recommend that experienced urological surgeons adopt this novel suture method for larger tumors when the clamping times extend limited ischemia duration.

There are a few limitations to this study. Firstly, more cases should be integrated into the following analysis to improve statistical power. Secondly, this study is a single-center and single-surgeon series. Thirdly, a long-term evaluation of renal function outcomes is absent.

## Conclusion

Our study suggests that the modified suturing method during transperitoneal LPN is safe and feasible for T1 renal tumor treatment, which greatly reduces WIT and preserves early postoperative kidney function. However, additional prospective studies and a longer follow-up period need to be performed.

## Data Availability Statement

The original contributions presented in the study are included in the article/supplementary material, further inquiries can be directed to the corresponding author.

## Ethics Statement

The studies involving human participants were reviewed and approved by Medical Ethics Committee of Shandong Provincial Hospital Affiliated to Shandong First Medical University. The patients/participants provided their written informed consent to participate in this study.

## Author Contributions

YJ: conception and design of the research and drafting the manuscript. HX: analysis and interpretation of data. QX: statistical analysis. QZ: revision of manuscript for important intellectual content. All authors have read and approved the manuscript and ensured that this is the case.

## Funding

This project is supported by the National Youth Fund of Natural Science Foundation (No. 81902572).

## Conflict of Interest

The authors declare that the research was conducted in the absence of any commercial or financial relationships that could be construed as a potential conflict of interest.

## Publisher's Note

All claims expressed in this article are solely those of the authors and do not necessarily represent those of their affiliated organizations, or those of the publisher, the editors and the reviewers. Any product that may be evaluated in this article, or claim that may be made by its manufacturer, is not guaranteed or endorsed by the publisher.
